# Epidemiology of Danish *Aeromonas salmonicida* subsp. *salmonicida* in Fish Farms Using Whole Genome Sequencing

**DOI:** 10.3389/fmicb.2017.02411

**Published:** 2017-12-05

**Authors:** Simona Bartkova, Pimlapas Leekitcharoenphon, Frank M. Aarestrup, Inger Dalsgaard

**Affiliations:** ^1^Section for Bacteriology and Pathology, National Veterinary Institute, Technical University of Denmark, Lyngby, Denmark; ^2^Research Group for Genomic Epidemiology, National Food Institute, Technical University of Denmark, Lyngby, Denmark

**Keywords:** *Aeromonas salmonicida* subsp. *salmonicida*, furunculosis, rainbow trout, whole genome sequencing, SNP analysis, BEAST, virulence factors

## Abstract

Furunculosis, a serious infection caused by the bacterium *Aeromonas salmonicida* subsp. *salmonicida* is common in sea-reared rainbow trout production in Denmark. Developing an effective control strategy requires knowledge of the epidemiology, as well as the genomic and virulent variability of the Danish *A. salmonicida* subsp. *salmonicida* isolates. To obtain this, the genomes of 101 *A. salmonicida* subsp. *salmonicida*, including 99 Danish isolates, one Scottish strain and the type strain NCIMB 1102, were sequenced using the Illumina HiSeq platform. Isolates were *de novo* assembled, examined for presence of plasmids, virulence and iron acquisition proteins, genomic islands, and antibiotic resistance genes. Single Nucleotide Polymorphisms were aligned and subjected to Bayesian temporal phylogenetic and maximum likelihood tree reconstruction using the published genome of *A. salmonicida* subsp. *salmonicida* A449 as reference. Bayesian temporal phylogenetic reconstruction suggests that four major introductions of *A. salmonicida* subsp. *salmonicida* into Denmark have occurred. The introductions correlate with the freshwater and subsequent seawater expansion of rainbow trout production. Initial transmission of the bacterium could have been from seawater to freshwater or vice versa, and most minor clades include a mixture of strains from different fresh- and seawater farms. Genomic variation of *A. salmonicida* subsp. *salmonicida* mostly appeared to be associated with their plasmids and plasmid encoded virulence factors. Nine *A. salmonicida* subsp. *salmonicida* isolates harbored worldwide known antibiotic resistance genes against several antibiotics and there is an indication that 33% of the isolates contained the genomic island AsaGEI1b. These findings not only support the usefulness of whole genome sequencing for genetic studies of homogeneous bacteria in general, but provide novel information about the Danish *A. salmonicida* subsp. *salmonicida* population, with implications for vaccine development in efforts to better protect Danish rainbow trout in the future.

## Introduction

*Aeromonas salmonicida* subsp. *salmonicida*, the causative agent of a serious infection furunculosis, was first isolated by Emmerich and Weibel (1894) at a German freshwater brown trout hatchery. Although the first rainbow trout (*Oncorhynchus mykiss*) hatchery in Denmark was already established in 1858 (Christensen, 1980), signs of furunculosis among fish were first described in the 1950s at freshwater rainbow trout farms (Rasmussen, 1964). At this point the Danish freshwater rainbow trout production had begun its massive expansion. In the late 1970s, production was extended to seawater and dry pellet feed was introduced instead of the common wet feed consisting of herring, whiting, sand-eels and other marine fish not used for human consumption (Christensen, 1980). Both actions increased the growth of the Danish rainbow trout production even further.

Currently, it is in the expanded Danish sea-reared rainbow trout production that *A. salmonicida* subsp. *salmonicida* is responsible for great financial losses. Despite fish being vaccinated before transfer from freshwater to seawater farms, furunculosis has occurred repeatedly during situations with elevated water temperatures (Larsen and Mellergaard, 1981; Dalsgaard and Madsen, 2000; Pedersen et al., 2008). This situation, along with previous research, has led to the belief that *A. salmonicida* subsp. *salmonicida* could be spread from freshwater to the sea by carrier fish that harbor the bacterium without showing any signs of disease (Larsen and Mellergaard, 1981; Dalsgaard and Madsen, 2000). Verifying this would be critical for developing an effective prevention strategy against furunculosis.

There are several genetic aspects of *A. salmonicida* subsp. *salmonicida* that could be used for epidemiological purposes and possibly aid in finding an effective prevention strategy against this bacterial species (e.g., Burr et al., 2002; Rasch et al., 2007; Reith et al., 2008; Dallaire-Dufresne et al., 2014; Vincent et al., 2014, 2015; Emond-Rheault et al., 2015a,b). First off, numerous potential virulence factors have been identified in *A. salmonicida* subsp. *salmonicida*, including extracellular proteases, lipases, adhesins, and functional secretion systems (Burr et al., 2002; Rasch et al., 2007; Reith et al., 2008; Dallaire-Dufresne et al., 2014). Iron acquisition has also been proven to be an important factor for virulence in almost all bacterial pathogens, including *A. salmonicida* subsp. *salmonicida* where it also seems to be linked to survival in aquatic environments (Reith et al., 2008) and to the innate immune response in the host (Ganz, 2009; Lee et al., 2014).

Certain virulence factors such as the A-layer (mainly composed of the VapA protein) in *A. salmonicida* subsp. *salmonicida* have a high antigenic conservation due to the A-layer surface structures being in contact with host defenses (Chart et al., 1984) and are encoded on the chromosome. Other virulence factors that are encoded on plasmids have shown a much greater diversity among *A. salmonicida* subsp. *salmonicida* (Nash et al., 2006; Vincent et al., 2015, 2016b). Among such belongs the type three secretion system (TTSS) encoded on the plasmid pAsa5 (Stuber et al., 2003; Reith et al., 2008), while the *aopP* gene that codes for a TTSS effector is present on the plasmid pAsal1 (Fehr et al., 2006). In general, the plasmid content of *A. salmonicida* subsp. *salmonicida* has shown to be variable and many new plasmids have been discovered in recent years (Boyd et al., 2003; Fehr et al., 2006; Reith et al., 2008; Vincent et al., 2014, 2016a; Attéré et al., 2015). Along with new plasmids, more antibiotic resistance genes (ARGs), some of which were previously only thought to be present in other bacterial species and ecological niches, have also been found on *A. salmonicida* subsp. *salmonicida* plasmids (R plasmids) (e.g., L'Abée-Lund and Sørum, 2001; Sørum et al., 2003; Kadlec et al., 2011; Vincent et al., 2014, 2016a).

Genomic islands (GEI) are another genetic element that has shown to vary among the *A. salmonicida* subsp. *salmonicida* population (Emond-Rheault et al., 2015a,b; Long et al., 2016). Emond-Rheault et al. (2015a) identified a new GEI named *Aeromonas salmonicida* genomic island (*AsaGEI*) in *A. salmonicida* subsp. *salmonicida*. Thus far this GEI has been found in *A. salmonicida* subsp. *salmonicida* in five different forms AsaGEI (1a, 1b, 2a, 2b, and 2c) varying in size from 50 to 53 kb, whose presence in *A. salmonicida* subsp. *salmonicida* seem to correlate with specific geographical origins of the bacterial isolates (Emond-Rheault et al., 2015a,b; Long et al., 2016).

To have an overview of the local epidemiology and evolution, as well as the variation in genomics and virulence factors of Danish *A. salmonicida* subsp. *salmonicida* isolates, a representative collection of 99 Danish *A. salmonicida* subsp. *salmonicida* isolates varying in isolation years 1980–2014 and geographical regions, a Scottish strain and the type strain NCIMB 1102 were sequenced using the Illumina HiSeq platform. Sequences of all isolates were *de novo* assembled and analyzed using the published genome of *A. salmonicida* subsp. *salmonicida* A449 (Reith et al., 2008) as reference.

## Methods

### Bacterial isolates

Ninety-Nine Danish *A. salmonicida* subsp. *salmonicida* isolates from furunculosis outbreaks between 1980 and 2014 were selected. The collection consisted of 42 *A. salmonicida* subsp. *salmonicida* isolated from various freshwater farms, of which 40 were from rainbow trout and two from brown trout (*Salmo trutta*). Fifty-seven of the *A. salmonicida* subsp. *salmonicida* were from rainbow trout at various seawater farms, including 14 isolates (isolated between 1981 and 2014) that belonged to one large seawater farm and nine isolates (isolated between 1989 and 2010) to another large seawater farm named Sj4 and Sj3 respectively in this study. All *Aeromonas salmonicida* subsp. *salmonicida* isolates were from diseased fish. The pigment of the colonies isolated from the fish was noted and the adherent “rough” colonies were selected confirmed to belong to the subspecies *salmonicida* according to methods by Dalsgaard and Madsen (2000). All the isolates were stored in broth culture supplemented with 15–20% glycerol at −80°C for long-term storage (Dalsgaard and Madsen, 2000). The Scottish *A. salmonicida* subsp. *salmonicida* strain MT004 from Atlantic salmon (*Salmo salar* L.) is according to literature isolated around 1980. The *A. salmonicida* subsp. *salmonicida* type strain NCIMB 1102 from England was isolated from an Atlantic salmon in year 1962. Extracted genomic DNA from all 101 *A. salmonicida* subsp. *salmonicida* was used for sequencing.

### Sample preparation

When taken out from their long-term storage, all *A. salmonicida* subsp. *salmonicida* were grown in Veal Infusion Broth (VIB) (Difco) at 20°C for 48 h and then inoculated on blood agar plates (Colombia agar base (Oxoid) with 5% calf blood at 20°C for 48–72 h. The blood agar plates were checked for haemolysis (clearing zone around the colonies) as well as shape of the colonies. Genomic DNA was extracted from bacterial colonies using a QIAGEN QIAamp DNA mini kit (QIAGEN, Valencia, CA, USA) according to the manufacturer's protocol. DNA quality was determined by NanoDrop ND-1000 (Thermo Scientific, Waltham, MA, USA) and DNA concentration by Qubit 2.0 fluorometer and Quant-iT dsDNA BR kit (Invitrogen, Carlsbad, CA, USA). All DNA extractions were immediately stored at −20°C until further use.

### Whole genome sequencing, *de novo* assembly, and antibiotic resistance genes

Genomic DNA was prepared for Illumina pair-end sequencing using the Illumina (Illumina, Inc., San Diego, CA) NexteraXT® Guide 150319425031942 following the protocol revision C (http://support.illumina.com/downloads/nextera_xt_sample_preparation_guide_15031942.html). A sample of the pooled NexteraXT Libraries was loaded onto an Illumina HiSeq reagent cartridge using HiSeq Reagent Kit v2 and 500 cycles with a Standard Flow Cell. The libraries were sequenced using an Illumina platform and HiSeq Control Software 2.3.0.3. The genomic DNA of all isolates was pair-end sequenced. Raw sequence data have been submitted to the European Nucleotide Archive (http://www.ebi.ac.uk/ena) under study accession no.: PRJEB15222. The raw reads were *de novo* assembled using the assemble pipeline (version 1.0) available from the Center for Genomic Epidemiology (CGE) https://cge.cbs.dtu.dk/services/Assembler/ which is based on the Velvet algorithms for *de novo* short reads assembly (Zerbino and Birney, 2008). Full genomic data can be retrieved from the Table S1.

Identification of acquired antibiotic resistance genes (ARGs) was performed through assembled genomes using the pipeline ResFinder (version 2.1) (Zankari et al., 2012) available from Center for Genomic Epidemiology (http://cge.cbs.dtu.dk/services/). Threshold for presence of an ARG in an isolate was set to 75% similarity expressed as percent sequence identity (ID) and 60% of alignment length (coverage) of resistance gene.

### Single nucleotide polymorphisms (SNPs)

SNPs were determined by pipeline; CSI phylogeny (Leekitcharoenphon et al., 2012; Kaas et al., 2014*)* available on the CGE (www.genomicepidemiology.org). The paired-end reads were mapped to the reference chromosome of the French *A. salmonicida* subsp. *salmonicida* strain A449 isolated year 1975 from a brown trout (accession number CP000644, chromosome length 4,702,402 bp) using Burrows-Wheeler Aligner (BWA) version 0.7.2 (Li and Durbin, 2009). The “mpileup” module in SAMTools version 0.1.18 (Li et al., 2009) was used to identify SNPs. Qualified SNPs were determined when fulfilling the following criteria: (1) a minimum distance of 10 bps between each SNP, (2) a minimum of 10% of the relative depth at SNP positions, (3) the mapping quality was above 25, (4) the SNP quality was more than 30 and (5) all indels were excluded. The SNPs from each genome were concatenated to a single alignment corresponding to position of the reference genome. The concatenated sequences were subjected to maximum likelihood tree using FasTree (Price et al., 2009).

### Temporal Bayesian phylogenetic tree

SNPs were used in Bayesian temporal phylogenetic reconstruction using BEAST (Bayesian Evolutionary Analysis Sampling Trees) version 1.7 (Drummond and Rambaut, 2007; Drummond et al., 2012) to estimate mutation rate and divergence time. Combinations of population size change and molecular clock were evaluated to identify the best-fit model (exponential clock and coalescent Bayesian skyline). The Bayesian temporal tree was constructed using the best-fit model. The BEAST MCMC chains were simulated for 300 million steps and subsampled every 10,000 steps. The final single maximum clade credibility (MCC) was examined using TreeAnnotator (Drummond et al., 2012) with 10% of the MCMC steps discarded as burn-in. Statistical confidence was represented by the 95% highest posterior density (HPD) interval.

### Virulence and iron acquisition proteins

The presence of virulence and iron acquisition proteins among all *A. salmonicida* subsp. *salmonicida* isolates was analyzed using blastp search (Altschul et al., 1990) with 78 known virulence associated and iron acquisition protein sequences (Table S2) found in the NCBI protein database against the assembled *A. salmonicida* subsp. *salmonicida* genomes. Threshold limit for presence of protein in an isolate was set to 65% ID and 60% coverage of protein.

### Plasmid and genomic island profiles

To obtain an indication of the plasmid and GEI content of each *A. salmonicida* subsp. *salmonicida*, a blastn search (Altschul et al., 1990) was performed with 17 known *A. salmonicida* subsp. *salmonicida* plasmids and five GEI sequences respectively, found in the NCBI database (Table S3) against the assembled *A. salmonicida* subsp. *salmonicida* genomes. Threshold limit for indicated presence of plasmid in an isolate was set to 75% ID and 60% coverage of plasmid (except for plasmids pAsa6 and pAsa7) due to the long length of plasmid. For plasmids pAsa6 and pAsa7 the coverage was increased to 100% coverage of plasmid, since pAsa6 is almost identical to the larger plasmid pAsa5 (Najimi et al., 2009) and pAsa7 to pAsa2 (Vincent et al., 2015). Threshold limit for indicated presence of GEIs in an isolate was set to 75% ID and 100% coverage due to the high sequence similarity of the GEIs.

## Results

### Phylogeny

**Figure 1:**
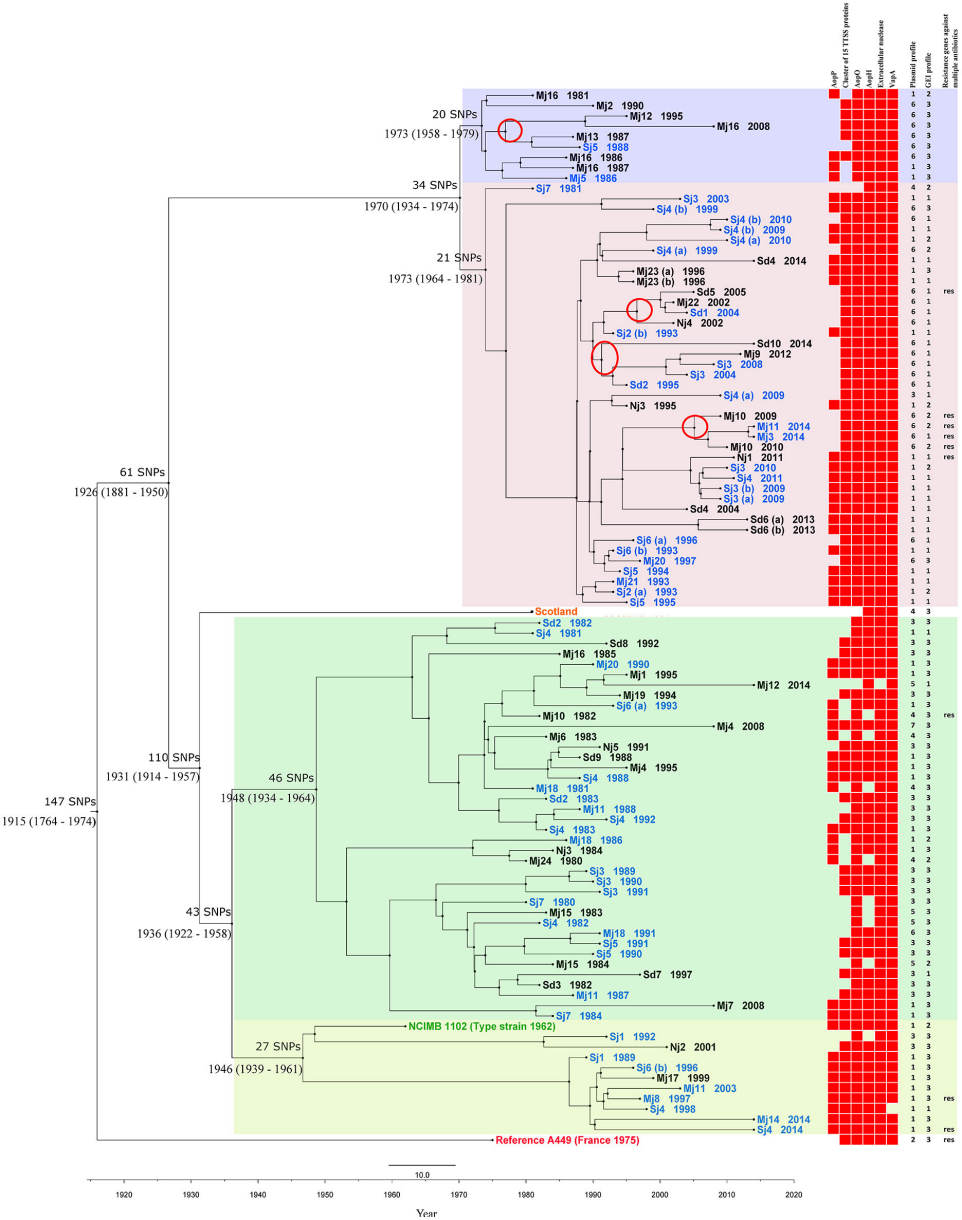
Phylogeny of *A. salmonicida* subsp. *salmonicida*. Bayesian temporal phylogenetic tree based on the alignment of 667 SNPs found among the 101 *A. salmonicida* subsp. *salmonicida* sequenced isolates and the reference A449. The tree shows the most recent common ancestor of the *A. salmonicida* subsp. *salmonicida* isolates dates back to ~1915 (95% HPD interval 1764–1947) and that there have been four main introductions of *A. salmonicida* subsp. *salmonicida* in Denmark: ~1973 (95% HPD interval 1958–1979), ~1973 (95% HPD interval 1964–1981), ~1948 (95% HPD interval 1934–1964) and ~1946 (95% HPD interval 1939–1961). The four main clades are each shaded with a color SNP differences between major clades are shown above the estimated year of emergence. The three non-Danish *A. salmonicida* subsp. *salmonicida* each have their own color and have the following labels: Scotland, NCIMB 1102 (type strain 1962), Reference A449 (France 1975). The Danish isolates either have a black color (freshwater farms) or a blue color (seawater farms) and they are labeled by region of origin followed by year of isolation. Following abbreviations are used for regions in Denmark: Nj, Northern Jutland; Mj, Central Jutland; Sd, Southern Denmark; Sj, Zealand. A heatmap illustration with information regarding acquired virulence and iron acquisition proteins, ARGs and plasmid and GEI profile numbers of each *A. salmonicida* subsp. *salmonicida* isolate is shown to the right of the tree. Presence and absence of protein sequences are illustrated by presence and absence of a red square, respectively. Plasmid profile number is shown and isolates that harbor ARGs against multiple antibiotics are labeled with “res.” Four minor clades marked with a red ring consist solely of isolates without plasmid pAsa3 and pAsal1.

In the chromosome of all the studied *A. salmonicida* subsp. *salmonicida* isolates, including the French reference strain A449, a total of 667 SNPs were identified and a SNP matrix was generated, illustrating the SNP difference between each isolate (Table S4). On average, there was a difference of 147 SNPs between the reference strain A449 and the rest of the *A. salmonicida* subsp. *salmonicida* isolates. When comparing the average SNP difference of the Scottish isolate and the type strain NCIMB 1102 to the rest of the isolates, the average difference was 115 and 41 SNPs respectively. The two Danish *A. salmonicida* subsp. *salmonicida* (Mj2 1990 and Sd8 1992), which were the only isolates from Denmark that were isolated from brown trout instead of rainbow trout, had an average difference of 50 and 42 SNPs respectively when compared to the rest of the isolate collection. The average difference among all the 99 sequenced Danish isolates was 47 SNPs among the Danish isolates from freshwater versus 46 SNPs among the isolates from seawater. The three Danish isolates with the highest average SNP difference were Sj7 1980 with 92 SNPs, Mj12 2014 with 67 SNPs and Mj4 2008 with 61 SNPs (Table S4). Based on the alignment of the 667 SNPs, two trees were constructed: a Bayesian temporal tree (Figure 1) with a Baysian Skyline population size change and an exponential clock rate as the best fit combination model for the *A. salmonicida* subsp. *salmonicida* population and a maximum likelihood tree (Figure S1) for topology confirmation. The two trees showed similar topology and the Bayesian tree (Figure 1) was illustrated with obtained genetic information regarding acquired ARGs, virulence and iron acquisition proteins and plasmid and GEI profiles of each *A. salmonicida* subsp. *salmonicida* isolate for further analysis.

The mutation rate of *A. salmonicida* subsp. *salmonicida* isolates was estimated to be 1.93 × 10^−7^ substitutions/site/year, which corresponds to 0.91 SNPs/genome/year. The most recent common ancestor of the *A. salmonicida* subsp. *salmonicida* isolates was estimated to have emerged in ~1915 (95% HPD interval 1764–1947). There are two major clades originating back to ~1926 (95% HPD interval 1881–1950) that each branched out further into two more clades in ~1936 (95% HPD interval 1922–1958) and ~1970 (95% HPD interval 1934–1974) respectively, resulting in roughly four main introductions of *A. salmonicida* subsp. *salmonicida* in Denmark: ~1973 (95% HPD interval 1958–1979), ~1973 (95% HPD interval 1964–1981), ~1948 (95% HPD interval 1934–1964), and ~1946 (95% HPD interval 1939–1961). From approximately 1975–1995 the Danish *A. salmonicida* subsp. *salmonicida* population experienced a massive clonal expansion. There was a correlation of local geographical transmission among the Danish freshwater isolates grouped together in the upper clade of the tree. There was another transmission link between isolates from a freshwater farm Mj10 and isolates from two seawater farms that had received fish from this farm.

### Antibiotic resistance

All sequenced *A. salmonicida* subsp. *salmonicida* isolates harbored three ARGs against beta-lactam antibiotics encoded on the chromosome (Table 1). Nine Danish *A. salmonicida* subsp. *salmonicida* isolates also harbored several other plasmid encoded resistance genes against trimethoprim, sulphonamide and aminoglycoside antibiotics (Tables 1, 2). All three isolates from freshwater farm Mj10 sampled during different years harbored ARGs against several of the above-mentioned antibiotics. The same ARGs against multiple antibiotics were found in isolates sampled from three seawater farms (Mj8 1997, Mj11 2014, and Mj3 2014) located in the same bay that all received fish from the freshwater farm Mj10. The French reference strain A449 also harbored ARGs against beta-lactam, sulphonamide, aminoglycoside, phenicol and tetracycline antibiotics and more resistance genes are described by Reith et al. (2008).

**Table 1:** Overview of acquired antibiotic resistance genes among the 101 *A. salmonicida* subsp. *salmonicida* sequenced isolates and the reference A449.

***A. salmonicida* subsp. *salmonicida* isolate**	**Beta-lactam**			**Trimethoprim**		**Sulphonamide**		**Aminoglycoside**				**Phenicol**	**Tetracycline**
Reference A449 (France 1975)	*blaFOX-2*	*ampS*	*blaCEPH-A3*	–	–	*sul1*	–	–	–	*aadA1*	–	*cat*	*tet(E)*
Sd5 2005	*blaFOX-2*	*ampS*	*blaCEPH-A3*	–	*dfrA1*	*sul1*	–	–	–	*aadA1*	–	–	–
Mj10 2009	*blaFOX-2*	*ampS*	*blaCEPH-A3*	–	*dfrA1*	*sul1*	–	–	–	*aadA1*	–	–	–
Mj10 2010	*blaFOX-2*	*ampS*	*blaCEPH-A3*	–	*dfrA1*	*sul1*	–	–	–	*aadA1*	–	–	–
Nj1 2011	*blaFOX-2*	*ampS*	*blaCEPH-A3*	–	*dfrA1*	*sul1*	–	–	*strB*	*aadA1*	*strA*	–	–
Mj11 2014	*blaFOX-2*	*ampS*	*blaCEPH-A3*	–	*dfrA1*	*sul1*	–	–	–	*aadA1*	–	–	–
Mj3 2014	*blaFOX-2*	*ampS*	*blaCEPH-A3*	–	*dfrA1*	*sul1*	–	–	–	*aadA1*	–	–	–
Sj4 2014	*blaFOX-2*	*ampS*	*blaCEPH-A3*	*dfrA14*	–	–	*sul2*	–	*strB*	–	*strA*	–	–
Mj10 1982	*blaFOX-2*	*ampS*	*blaCEPH-A3*	–	–	*sul1*	–	*aadA2*	–	–	–	–	–
Mj8 1997	*blaFOX-2*	*ampS*	*blaCEPH-A3*	–	*dfrA1*	*sul1*	–	–	–	*aadA1*	–	–	–
Nj2 2001	*blaFOX-2*	*ampS*	*blaCEPH-A3*	–	–	–	–	–	–	–	–	–	–
Mj22 2002	*blaFOX-2*	*ampS*	*blaCEPH-A3*	–	–	–	–	–	–	–	–	–	–
Nj4 2002	*blaFOX-2*	*ampS*	*blaCEPH-A3*	–	–	–	–	–	–	–	–	–	–
Mj11 2003	*blaFOX-2*	*ampS*	*blaCEPH-A3*	–	–	–	–	–	–	–	–	–	–
Sj3 2003	*blaFOX-2*	*ampS*	*blaCEPH-A3*	–	–	–	–	–	–	–	–	–	–
Sd1 2004	*blaFOX-2*	*ampS*	*blaCEPH-A3*	–	–	–	–	–	–	–	–	–	–
Sd4 2004	*blaFOX-2*	*ampS*	*blaCEPH-A3*	–	–	–	–	–	–	–	–	–	–
Sj3 2004	*blaFOX-2*	*ampS*	*blaCEPH-A3*	–	–	–	–	–	–	–	–	–	–
Mj7 2008	*blaFOX-2*	*ampS*	*blaCEPH-A3*	–	–	–	–	–	–	–	–	–	–
Mj16 2008	*blaFOX-2*	*ampS*	*blaCEPH-A3*	–	–	–	–	–	–	–	–	–	–
Mj4 2008	*blaFOX-2*	*ampS*	*blaCEPH-A3*	–	–	–	–	–	–	–	–	–	–
Sj3 2008	*blaFOX-2*	*ampS*	*blaCEPH-A3*	–	–	–	–	–	–	–	–	–	–
Sj4 (a) 2009	*blaFOX-2*	*ampS*	*blaCEPH-A3*	–	–	–	–	–	–	–	–	–	–
Sj3 (a) 2009	*blaFOX-2*	*ampS*	*blaCEPH-A3*	–	–	–	–	–	–	–	–	–	–
Sj3 (b) 2009	*blaFOX-2*	*ampS*	*blaCEPH-A3*	–	–	–	–	–	–	–	–	–	–
Sj4 (b) 2009	*blaFOX-2*	*ampS*	*blaCEPH-A3*	–	–	–	–	–	–	–	–	–	–
Sj3 2010	*blaFOX-2*	*ampS*	*blaCEPH-A3*	–	–	–	–	–	–	–	–	–	–
Sj4 (a) 2010	*blaFOX-2*	*ampS*	*blaCEPH-A3*	–	–	–	–	–	–	–	–	–	–
Sj4 (b) 2010	*blaFOX-2*	*ampS*	*blaCEPH-A3*	–	–	–	–	–	–	–	–	–	–
Sj4 2011	*blaFOX-2*	*ampS*	*blaCEPH-A3*	–	–	–	–	–	–	–	–	–	–
Mj9 2012	*blaFOX-2*	*ampS*	*blaCEPH-A3*	–	–	–	–	–	–	–	–	–	–
Mj12 2014	*blaFOX-2*	*ampS*	*blaCEPH-A3*	–	–	–	–	–	–	–	–	–	–
Sd6 (a) 2013	*blaFOX-2*	*ampS*	*blaCEPH-A3*	–	–	–	–	–	–	–	–	–	–
Sd6 (b) 2013	*blaFOX-2*	*ampS*	*blaCEPH-A3*	–	–	–	–	–	–	–	–	–	–
Mj14 2014	*blaFOX-2*	*ampS*	*blaCEPH-A3*	–	–	–	–	–	–	–	–	–	–
Sd4 2014	*blaFOX-2*	*ampS*	*blaCEPH-A3*	–	–	–	–	–	–	–	–	–	–
Sd10 2014	*blaFOX-2*	*ampS*	*blaCEPH-A3*	–	–	–	–	–	–	–	–	–	–
Sj7 1980	*blaFOX-2*	*ampS*	*blaCEPH-A3*	–	–	–	–	–	–	–	–	–	–
Mj24 1980	*blaFOX-2*	*ampS*	*blaCEPH-A3*	–	–	–	–	–	–	–	–	–	–
Mj18 1981	*blaFOX-2*	*ampS*	*blaCEPH-A3*	–	–	–	–	–	–	–	–	–	–
Sj7 1981	*blaFOX-2*	*ampS*	*blaCEPH-A3*	–	–	–	–	–	–	–	–	–	–
Sj4 1981	*blaFOX-2*	*ampS*	*blaCEPH-A3*	–	–	–	–	–	–	–	–	–	–
Mj16 1981	*blaFOX-2*	*ampS*	*blaCEPH-A3*	–	–	–	–	–	–	–	–	–	–
Sd3 1982	*blaFOX-2*	*ampS*	*blaCEPH-A3*	–	–	–	–	–	–	–	–	–	–
Sj4 1982	*blaFOX-2*	*ampS*	*blaCEPH-A3*	–	–	–	–	–	–	–	–	–	–
Sd2 1982	*blaFOX-2*	*ampS*	*blaCEPH-A3*	–	–	–	–	–	–	–	–	–	–
Sj4 1983	*blaFOX-2*	*ampS*	*blaCEPH-A3*	–	–	–	–	–	–	–	–	–	–
Mj6 1983	*blaFOX-2*	*ampS*	*blaCEPH-A3*	–	–	–	–	–	–	–	–	–	–
Mj15 1983	*blaFOX-2*	*ampS*	*blaCEPH-A3*	–	–	–	–	–	–	–	–	–	–
Sd2 1983	*blaFOX-2*	*ampS*	*blaCEPH-A3*	–	–	–	–	–	–	–	–	–	–
Sj7 1984	*blaFOX-2*	*ampS*	*blaCEPH-A3*	–	–	–	–	–	–	–	–	–	–
Mj15 1984	*blaFOX-2*	*ampS*	*blaCEPH-A3*	–	–	–	–	–	–	–	–	–	–
Nj3 1984	*blaFOX-2*	*ampS*	*blaCEPH-A3*	–	–	–	–	–	–	–	–	–	–
Mj16 1985	*blaFOX-2*	*ampS*	*blaCEPH-A3*	–	–	–	–	–	–	–	–	–	–
Mj5 1986	*blaFOX-2*	*ampS*	*blaCEPH-A3*	–	–	–	–	–	–	–	–	–	–
Mj18 1986	*blaFOX-2*	*ampS*	*blaCEPH-A3*	–	–	–	–	–	–	–	–	–	–
Mj16 1986	*blaFOX-2*	*ampS*	*blaCEPH-A3*	–	–	–	–	–	–	–	–	–	–
Mj11 1987	*blaFOX-2*	*ampS*	*blaCEPH-A3*	–	–	–	–	–	–	–	–	–	–
Mj16 1987	*blaFOX-2*	*ampS*	*blaCEPH-A3*	–	–	–	–	–	–	–	–	–	–
Mj13 1987	*blaFOX-2*	*ampS*	*blaCEPH-A3*	–	–	–	–	–	–	–	–	–	–
Sj5 1988	*blaFOX-2*	*ampS*	*blaCEPH-A3*	–	–	–	–	–	–	–	–	–	–
Sj4 1988	*blaFOX-2*	*ampS*	*blaCEPH-A3*	–	–	–	–	–	–	–	–	–	–
Sd9 1988	*blaFOX-2*	*ampS*	*blaCEPH-A3*	–	–	–	–	–	–	–	–	–	–
Mj11 1988	*blaFOX-2*	*ampS*	*blaCEPH-A3*	–	–	–	–	–	–	–	–	–	–
Sj1 1989	*blaFOX-2*	*ampS*	*blaCEPH-A3*	–	–	–	–	–	–	–	–	–	–
Sj3 1989	*blaFOX-2*	*ampS*	*blaCEPH-A3*	–	–	–	–	–	–	–	–	–	–
Mj2 1990	*blaFOX-2*	*ampS*	*blaCEPH-A3*	–	–	–	–	–	–	–	–	–	–
Sj3 1990	*blaFOX-2*	*ampS*	*blaCEPH-A3*	–	–	–	–	–	–	–	–	–	–
Mj20 1990	*blaFOX-2*	*ampS*	*blaCEPH-A3*	–	–	–	–	–	–	–	–	–	–
Sj5 1990	*blaFOX-2*	*ampS*	*blaCEPH-A3*	–	–	–	–	–	–	–	–	–	–
Scotland	*blaFOX-2*	*ampS*	*blaCEPH-A3*	–	–	–	–	–	–	–	–	–	–
Sj3 1991	*blaFOX-2*	*ampS*	*blaCEPH-A3*	–	–	–	–	–	–	–	–	–	–
Nj5 1991	*blaFOX-2*	*ampS*	*blaCEPH-A3*	–	–	–	–	–	–	–	–	–	–
Sj5 1991	*blaFOX-2*	*ampS*	*blaCEPH-A3*	–	–	–	–	–	–	–	–	–	–
Mj18 1991	*blaFOX-2*	*ampS*	*blaCEPH-A3*	–	–	–	–	–	–	–	–	–	–
Sj4 1992	*blaFOX-2*	*ampS*	*blaCEPH-A3*	–	–	–	–	–	–	–	–	–	–
Sj1 1992	*blaFOX-2*	*ampS*	*blaCEPH-A3*	–	–	–	–	–	–	–	–	–	–
Sd8 1992	*blaFOX-2*	*ampS*	*blaCEPH-A3*	–	–	–	–	–	–	–	–	–	–
Sj6 (a) 1993	*blaFOX-2*	*ampS*	*blaCEPH-A3*	–	–	–	–	–	–	–	–	–	–
Sj6 (b) 1993	*blaFOX-2*	*ampS*	*blaCEPH-A3*	–	–	–	–	–	–	–	–	–	–
Sj2 (a) 1993	*blaFOX-2*	*ampS*	*blaCEPH-A3*	–	–	–	–	–	–	–	–	–	–
Sj2 (b) 1993	*blaFOX-2*	*ampS*	*blaCEPH-A3*	–	–	–	–	–	–	–	–	–	–
Mj21 1993	*blaFOX-2*	*ampS*	*blaCEPH-A3*	–	–	–	–	–	–	–	–	–	–
Mj19 1994	*blaFOX-2*	*ampS*	*blaCEPH-A3*	–	–	–	–	–	–	–	–	–	–
Sj5 1994	*blaFOX-2*	*ampS*	*blaCEPH-A3*	–	–	–	–	–	–	–	–	–	–
Nj3 1995	*blaFOX-2*	*ampS*	*blaCEPH-A3*	–	–	–	–	–	–	–	–	–	–
Mj12 1995	*blaFOX-2*	*ampS*	*blaCEPH-A3*	–	–	–	–	–	–	–	–	–	–
Mj1 1995	*blaFOX-2*	*ampS*	*blaCEPH-A3*	–	–	–	–	–	–	–	–	–	–
Mj4 1995	*blaFOX-2*	*ampS*	*blaCEPH-A3*	–	–	–	–	–	–	–	–	–	–
Sj5 1995	*blaFOX-2*	*ampS*	*blaCEPH-A3*	–	–	–	–	–	–	–	–	–	–
Sd2 1995	*blaFOX-2*	*ampS*	*blaCEPH-A3*	–	–	–	–	–	–	–	–	–	–
Mj23 (a) 1996	*blaFOX-2*	*ampS*	*blaCEPH-A3*	–	–	–	–	–	–	–	–	–	–
Mj23 (b) 1996	*blaFOX-2*	*ampS*	*blaCEPH-A3*	–	–	–	–	–	–	–	–	–	–
Sj6 (a) 1996	*blaFOX-2*	*ampS*	*blaCEPH-A3*	–	–	–	–	–	–	–	–	–	–
Sj6 (b) 1996	*blaFOX-2*	*ampS*	*blaCEPH-A3*	–	–	–	–	–	–	–	–	–	–
Sd7 1997	*blaFOX-2*	*ampS*	*blaCEPH-A3*	–	–	–	–	–	–	–	–	–	–
Mj20 1997	*blaFOX-2*	*ampS*	*blaCEPH-A3*	–	–	–	–	–	–	–	–	–	–
Sj4 1998	*blaFOX-2*	*ampS*	*blaCEPH-A3*	–	–	–	–	–	–	–	–	–	–
Sj4 (a) 1999	*blaFOX-2*	*ampS*	*blaCEPH-A3*	–	–	–	–	–	–	–	–	–	–
Mj17 1999	*blaFOX-2*	*ampS*	*blaCEPH-A3*	–	–	–	–	–	–	–	–	–	–
Sj4 (b) 1999	*blaFOX-2*	*ampS*	*blaCEPH-A3*	–	–	–	–	–	–	–	–	–	–
NCIMB 1102 (Type strain 1962)	*blaFOX-2*	*ampS*	*blaCEPH-A3*	–	–	–	–	–	–	–	–	–	–

*Threshold for presence of a resistance gene in an isolate was set to 75% similarity expressed as percent sequence identity (ID) and 60% of alignment length (coverage) of the resistance gene. Isolates are labeled according to region and year of isolation as in Figure 1*.

**Table 2:** Overview of plasmid profiles among the 101 *A. salmonicida* subsp. *salmonicida* sequenced isolates and the reference A449.

**Profile no**.	**No. of *A. salmonicida* subsp. *salmonicida* isolates**	**Plasmids**																
		**pAsa1**	**pAsa2**	**pAsa3**	**pAsa4**	**pAsa5**	**pAsa6**	**pAsa7**	**pAsa8**	**pAsal1**	**pAr_32**	**pRAS1**	**pRAS3.1**	**pRAS3.2**	**pRAS3.3**	**pSN254b**	**pAB5S9b**	**pY47**
1	46	+	+	+	–	+	–	–	–	+	–	–	–	–	–	–	–	–
2	1	+	+	+	+	+	–	–	–	–	–	–	–	–	–	–	–	–
3	20	+	+	+	–	+	–	–	–	–	–	–	–	–	–	–	–	-
4	6	+	+	+	–	–	–	–	–	+	–	–	–	–	–	–	–	–
5	4	+	+	+	–	–	–	–	–	–	–	–	–	–	–	–	–	–
6	24	+	+	–	–	+	–	–	–	–	–	–	–	–	–	–	–	–
7	1	+	–	+	–	+	–	–	–	+	–	–	–	–	–	–	–	–

*Plasmid profile number is displayed to the right, as well as the number of A. salmonicida subsp. salmonicida that have the respective profile. Presence and absence of a plasmid for the given profile is presented with a plus (present) and minus (absent) sign respectively, below each plasmid name*.

### Virulence and iron acquisition

**Figure 2:**
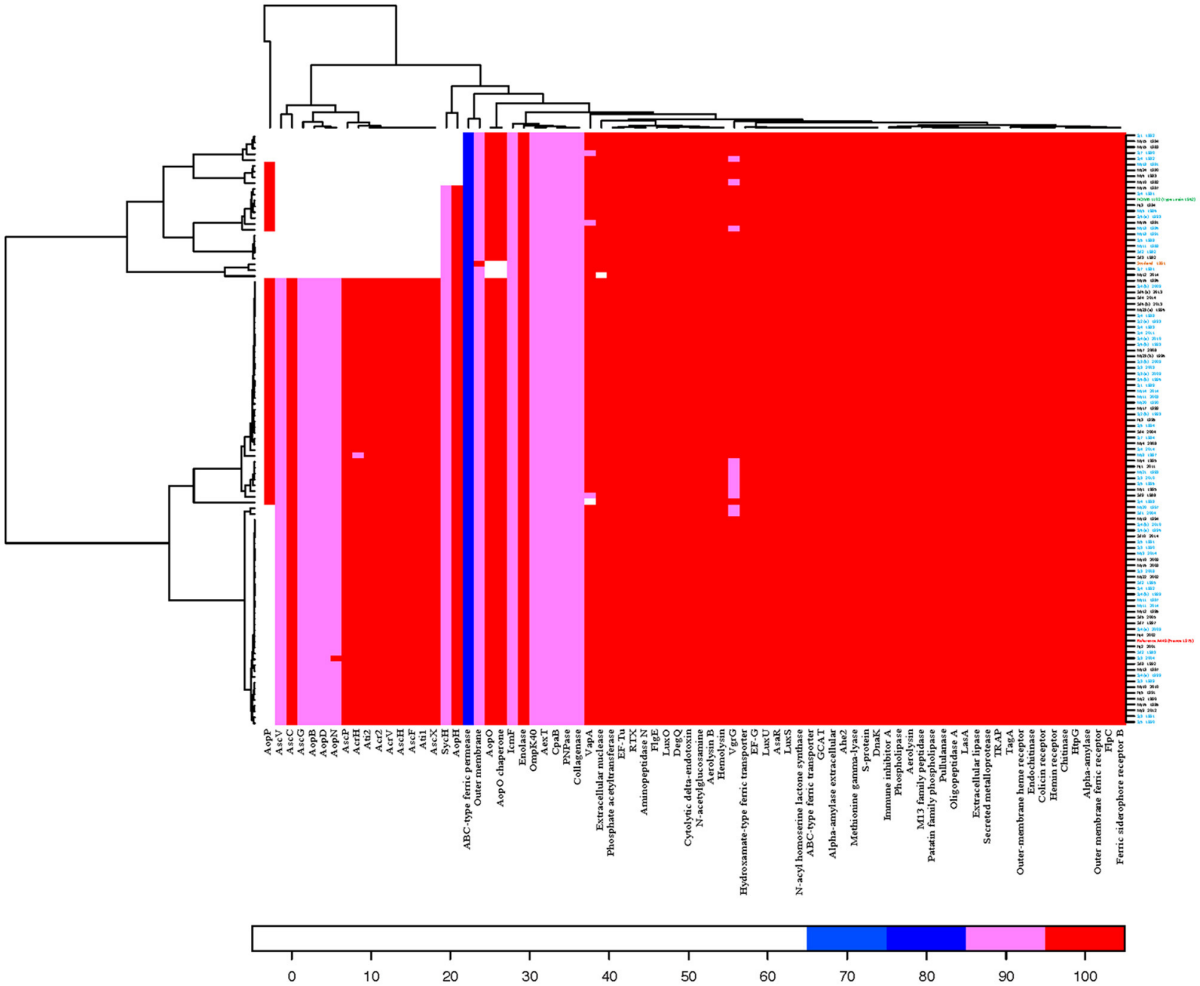
Heatmap illustrating presence and absence of 78 virulence associated and iron acquisition protein sequences found in the NCBI protein database among the 101 *A. salmonicida* subsp. *salmonicida* sequenced isolates and the reference A449. Isolates are displayed on the right and sequence protein names on the bottom. Threshold limit for presence of protein in an isolate was set to 65% similarity, expressed as percent sequence identity (ID) and 60% of alignment length (coverage) of the protein sequence. Red color represents > 95% ID, pink color > 85% ID, dark blue > 75% ID, and light blue > 65% ID.

Out of 78 investigated protein sequences, 22 were considered as absent (<65% ID) in one or more isolates (Figure 2). The T3SS effector protein AopP encoded on plasmid pAsal1 by the *aopP* gene was absent in 50% of the *A. salmonicida* subsp. *salmonicida* isolates, including the reference strain A449. A cluster of 15 T3SS related proteins were absent in 25 isolates. In nine of the 25 isolates, the T3SS putative tyrosine phosphatase AopH and its chaperone that are encoded on pAsa5 and have homologs encoded on pAsa6 were also absent. Three of the isolates were also missing the T3SS putative serine/threonine kinase AopO and its chaperone that are encoded on pAsa5, while isolate Mj12 2014 was missing the extracellular nuclease protein (48% ID) coded by the gene *nucH* on the chromosome. Isolate Sj4 1998, which is not included in above mentioned group of 25 isolates, did not possess the tetragonal surface virulence array protein VapA (A-layer) encoded on the chromosome. Lastly, the chromosome encoded ABC-type ferric siderophore transporter permease protein only showed 75% ID in all sequenced *A. salmonicida* subsp. *salmonicida* as well as the reference strain A449.

### Plasmid and genomic island profiles

Presence of multiple plasmids was indicated in all examined *A. salmonicida* subsp. *salmonicida* isolates (Table S5). Six plasmids seemed present in one or more isolates, while 11 plasmids were not found in any isolates. The only plasmid found in all isolates was pAsa1, although pAsa2 and pAsa5 showed high stability with an indicated presence of 99 and 90% respectively, among the isolates. The two plasmids pAsa3 and pAsal1 seemed present in 76 and 52% of the isolates respectively, while pAsa4 was only found in the reference strain A449. Seven different plasmid profiles were detected among the isolates, with one profile consisting of pAsa1, pAsa2, pAsa3, pAsa5, and pAsal1 representing 46% of the isolates (Table 2).

Ten plasmids are known for harboring ARGs (R plasmids): pAsa4, pAsa7, pAsa8, pAr-32, pRAS1, pRAS3.1, pRAS3.2, pRAS3.3, pSN254b, and pAB5S9b (Table S3) and only plasmid pAsa4 was found in any of the investigated *A. salmonicida* subsp. *salmonicida*.

All 51 *A. salmonicida* subsp. *salmonicida* isolates, in which the AopP protein encoded on pAsal1 was absent (Figure 2), were missing plasmid pAsal1 (Table S4). Of the 25 *A. salmonicida* subsp. *salmonicida* isolates that were missing a cluster of 15 T3SS proteins encoded on pAsa5, 10 also showed no presence of plasmid pAsa5. The remaining 15 isolates all displayed <80% coverage of the pAsa5. All nine *A. salmonicida* subsp. *salmonicida* isolates that lacked the protein AopH and its chaperone that are encoded on pAsa5 and have homologs on pAsa6 showed <80% coverage for pAsa5 and showed no indication of pAsa6 presence.

The *A. salmonicida* subsp. *salmonicida* isolates indicated a presence of three different GEI profiles (Table S6). Thirty-three percent of the isolates had profile number one, indicating presence of the GEI named AsaGEI1b. Fourteen percent of the isolates had a <80% coverage of AsaGEI1b, indicating a presence of a putative AsaGEI and were given the profile number two. The rest of the isolates had profile number three, as none of the five investigated GEIs were found in these isolates.

## Discussion

### Phylogeny

*A. salmonicida* subsp. *salmonicida* subspecies is known to be a highly homogeneous group that was considered clonal (Wiklund and Dalsgaard, 1998; Garcia et al., 2000; Cunningham and Colquhoun, 2002; Beaz-Hidalgo et al., 2008). The fact that only a total of 667 SNPs were found in the entire 4,702,402 bp long chromosome among the investigated *A. salmonicida* subsp. *salmonicida* varying in year of isolation (span of 34 years), geographical region, and host fish species only confirms this further. The highest average SNP difference (average of 147 SNPs) was found between the French reference strain A449 and the remaining *A. salmonicida* subsp. *salmonicida* isolates (Table S4), which is not a large difference considering that A449 was isolated in France and from a brown trout, while almost all (97 out of 99) of the Danish isolates were isolated from rainbow trout. When comparing the average SNP difference between the Scottish strain from Atlantic salmon and the rest of the isolates, the results were even lower with an average of 115 SNPs (Table S4). The two Danish *A. salmonicida* subsp. *salmonicida* isolated from brown trout (Mj2 1990 and Sd8 1992) and the type strain NCIMB 1102 from Atlantic salmon also grouped together with Danish *A. salmonicida* subsp. *salmonicida* from rainbow trout in one of the four major clades (Figure 1) and only have an average difference of 41, 50, and 52 SNPs, which challenges the theory of *A. salmonicida* subsp. *salmonicida* genome adapting to the environment of their specific hosts species (Reith et al., 2008). More *A. salmonicida* subsp. *salmonicida* isolates from various fish species would, however, need to be sequenced to shed more light on this theory.

Noticeably there appears to be four major *A. salmonicida* subsp. *salmonicida* introductions to Denmark, giving rise to four major clades (Figure 1). The two introductions that occurred in ~1973 (95% HPD interval 1958–1979) and ~1973 (95% HPD interval 1964–1981) and gave rise to the two upper clades (Figure 1), seemingly took place right before the massive clonal expansion during 1975–1995, which all four clades underwent. The two introduction points in 1973 and the expansion period of all four clades correspond to the time period where rainbow trout farming in Denmark began expanding out to seawater and intensifying their production (Christensen, 1980). The two bottom clades were introduced further in the past ~1948 (95% HPD interval 1934–1964) and ~1946 (95% HPD interval 1939–1961) respectively and contain *A. salmonicida* subsp. *salmonicida* with older isolation years (average year of isolation 1991), than the two upper clades that include *A. salmonicida* subsp. *salmonicida* with an average isolation year of 2001 (Figure 1).

The introduction of the two bottom clades ~1948 (95% HPD interval 1934–1964) and ~1946 (95% HPD interval 1939–1961) respectively, correlate with the end of the Second World War and the beginning of an expansion of rainbow trout production in Danish freshwater (Christensen, 1980). When examining the branches of each of the four major clades, there is a possibility that *A. salmonicida* subsp. *salmonicida* might have been introduced into freshwater through marine fish wet feed used until the late 1970s (Christensen, 1980). However, the bacterium could also have been transmitted from freshwater to seawater, which is the widespread theory i.e., that *A. salmonicida* subsp. *salmonicida* is present in freshwater fish showing no signs of diseases (carriers) and are then transferred out to seawater with the fish, where outbreaks occur during high temperatures (Larsen and Mellergaard, 1981; Dalsgaard and Madsen, 2000; Pedersen et al., 2008).

The local transmission pattern of *A. salmonicida* subsp. *salmonicida* among the Danish farms also suggests that transmission of isolates from freshwater to seawater farms have occurred, as exemplified by a minor clade where ARGs were transmitted from a freshwater farm to seawater farms. Isolates from different freshwater farms are moreover mixed with different isolates from seawater in most of the minor clades. Though, in general it is hard to find specific geographical correlations between the fish farms. One of the main causes for this could be the widespread trade of fingerlings for anglers in Denmark throughout the years as well as local trade among fish farmers. There is nonetheless a correlation among the group of freshwater farms isolates in the top clade. Mj13 is located upstream to Mj16 in a stream that runs out into a river named Guden Å. Two other farms (Mj12 and Mj2) are also located at streams that lead out to Guden Å and one of these (Mj12) produces brown trout.

### Antibiotic resistance

Interestingly, all investigated *A. salmonicida* subsp. *salmonicida* isolates possessed three beta-lactam ARGs (Table 1). Since the genes are encoded on the chromosome, it seems that either they have always been a part of the *A. salmonicida* subsp. *salmonicida* genome repertoire, or they must have been acquired at least around 67–250 years ago where they all have a most recent common ancestor (Figure 1). Nine Danish *A. salmonicida* subsp. *salmonicida* also harbored resistance genes against trimethoprim, sulphonamide and aminoglycoside antibiotics (Table 1), which are all plasmid encoded. Trimethoprim and sulphonamide are also two of the scarce number of antibiotics allowed to be used for treatment of bacterial diseases in Danish fish farms (Dalsgaard and Madsen, 2000). All three *A. salmonicida* subsp. *salmonicida* isolates from freshwater farm Mj10, isolated year 1982, 2009, and 2010 harbored resistance genes against at least two of the above-mentioned antibiotics (Table 1). From farm Mj10, fish have always been transferred out to a bay, where several seawater farms are located. In *A. salmonicida* subsp. *salmonicida* isolated during 2014 from two of the seawater farms in the bay (Figure 1), the same set of resistance genes were detected as those seen in *A. salmonicida* subsp. *salmonicida* from the freshwater farm Mj10 during 2009 and 2010 (Table 1). Fittingly, the two freshwater isolates form a minor clade with the two seawater isolates in the Bayesian temporal tree, according to which the isolates spread from the freshwater to seawater (Figure 1). In seawater farm Mj8, which is also located in the bay, an *A. salmonicida* subsp. *salmonicida* isolate from 1997 did harbor ARGs against the mentioned antibiotics as well. However, these ARGs were slightly different than those seen in the Mj10 1982 isolate, where one otherwise would expect the resistance genes had originated (Table 1). This could be associated with the fact that trimethoprim was not licensed for use in Denmark until 1983 (Dalsgaard and Madsen, 2000) and the isolate from 1982 had therefore not acquired ARGs against this antibiotic.

All nine of the above-mentioned isolates showed coverage (< 60%) of at least one of the R plasmids (Table S5), indicating they could have acquired ARGs from the plasmids in the past through horizontal gene transfer and then lost the plasmid. Isolate Sj4 2014 and Nj1 2011 were the only two isolates harboring ARGs *strA* and *strB*. Both ARGs are known to be present on the R plasmids pAB5S9b and pSN254b, but none of the investigated isolates in this study showed any coverage of these two plasmids. It is thus most likely that the isolates Sj4 2014 and Nj1 2011 carry the ARGs on new plasmids.

### Iron acquisition and virulence

When looking at the phylogeny, it was found that isolates grouped in the two bottom clades were missing a higher amount of virulence associated proteins (average of 1.0 protein per isolate), compared to isolates grouped in the two upper clades where the average absence of virulence associated proteins was 0.6 per isolate (Figure 1). This indicates that the most recent common ancestor of the bottom two clades from ~1936 (95% HPD interval 1922–1958), presumably harbored lower amount of virulence associated proteins and thus gave rise to two older lineages with fewer virulence associated proteins than the more recently introduced two lineages (the two upper clades in the phylogeny tree). Considering these results, it could be suggested that the upper two clades might consist of more virulent *A. salmonicida* subsp. *salmonicida* that were introduced from a more recent and more virulent ancestor around 1970.

Due to the important nature of iron acquisition for *A. salmonicida* subsp. *salmonicida* (Reith et al., 2008; Ganz, 2009; Lee et al., 2014) and the fact that all the investigated proteins related to iron acquisition were encoded on the chromosome, it was expected that all 101 sequenced *A. salmonicida* subsp. *salmonicida* isolates and the A449 reference genome possessed all the investigated iron acquisition proteins (Table S2). Though, it must be noted that the ABC-type ferric siderophore transporter permease protein only showed 75% ID in all 101 *A. salmonicida* subsp. *salmonicida* isolates as well as A449.

Most of potential virulence proteins investigated in this study were present in all sequenced *A. salmonicida* subsp. *salmonicida* and the reference strain. Though, two proteins encoded on the chromosome and 20 encoded on plasmids were missing in at least one isolate (Table S2). All the 20 plasmid encoded proteins were related to the functional type three secretion system (T3SS). This secretion system is wide spread among Gram-negative bacteria and has several functions, including: disrupting host cells by translocating toxins (effector proteins) into their cytoplasm, preventing phagocytosis by leukocytes, and establishing systemic infection (Burr et al., 2003, 2005; Stuber et al., 2003; Dacanay et al., 2006; Rasch et al., 2007; Dallaire-Dufresne et al., 2014). T3SS is also the only factor proven to be essential for virulence of *A. salmonicida* subsp. *salmonicida*, as all *in vitro* and *in vivo* studies involving inactivation of T3SS structural proteins in *A. salmonicida* subsp. *salmonicida* strains have resulted in non-virulent *A. salmonicida* subsp. *salmonicida* mutants (Burr et al., 2002, 2003, 2005; Stuber et al., 2003; Dacanay et al., 2006; Froquet et al., 2007). Nevertheless, among the 20 missing T3SS related proteins in this study were T3SS structural proteins, while all the *A. salmonicida* subsp. *salmonicida* in this study are isolated from furunculosis outbreaks, whereby one would assume that all the *A. salmonicida* subsp. *salmonicida* isolates are virulent.

There are 36 T3SS encoding genes located on the large plasmid pAsa5 (Reith et al., 2008; Najimi et al., 2009; Tanaka et al., 2012; Vincent et al., 2016b) and 19 of them that were investigated in this study were missing in at least three isolates (Table S2). Though, it is known that pAsa5 becomes unstable under stressful conditions like being subjected to growth in temperature above 22–25°C (Stuber et al., 2003; Tanaka et al., 2012; Dallaire-Dufresne et al., 2014) and is thought to undergo genetic rearrangement resulting in the loss of its T3SS region caused by activation of *ISAS11* insertion sequence (IS) elements (Tanaka et al., 2012). This could explain the fact that all the 25 *A. salmonicida* subsp. *salmonicida* isolates missing the cluster of T3SS proteins, encoded on pAsa5 in our study still harbored the plasmid, but displayed <80% coverage. The only issue with this justification is that all *A. salmonicida* subsp. *salmonicida* cultures in our laboratory are always grown at 20°C, meaning it is unlikely that growth at high temperature triggered the activation of IS*AS11*. It is unclear what other cause for the rearrangement of pAsa5 could have been.

Unlike most of the T3SS proteins, the AopP protein is encoded on plasmid pAsal1 and the protein was missing in 50% of the *A. salmonicida* subsp. *salmonicida* isolates investigated in this study (Figure 2). Interestingly, pAsal1 was present in isolate Sj 1981 and the Scottish isolate, both of which did not harbor the AopP protein. When analyzed using the program BioEdit (Hall, 1999), the isolates did possess the nucleotide sequence for the *aopP* gene; however, both sequences had an identical frameshift mutation caused by point deletions (data not showed) that presumably lead to an incorrect translation of the AopP protein sequence. Apart from the two mentioned isolates, all *A. salmonicida* subsp. *salmonicida* that were missing the AopP protein sequence were also missing the plasmid pAsal1. Previous studies have shown that pAsal1 is lost due to activation of the same insertion sequence as in pAsa5 (Daher et al., 2011; Tanaka et al., 2012; Attéré et al., 2015; Vincent et al., 2016a), though the plasticity of *A. salmonicida* subsp. *salmonicida* pAsal1 is complex, as the precise mechanism responsible for loss of pAsal1 remains unknown (Attéré et al., 2015) and at least three larger variants of the plasmid exist: pAsal1B, pAsal1C, and pAsal1D (Trudel et al., 2013; Attéré et al., 2015).

As illustrated on Figure 2, there was otherwise an overall high similarity among all isolates regarding all the chromosome encoded virulence associated protein sequences. The high prevalence and similarity of the A-layer (VapA) in *A. salmonicida* subsp. *salmonicida* discovered in this study, along with previous findings of *A. salmonicida* subsp. *salmonicida* surface structures being highly conserved (Chart et al., 1984), could also provide valuable knowledge for future vaccine development.

### Plasmid profiles

Out of the seven plasmid profiles found in this study, the most abundant profile consisting of pAsa1, pAsa2, pAsa3, pAsa5, and pAsal1 represented 46% of the *A. salmonicida* isolates (Table 2). In congruence, Nielsen et al. (1993) investigated *A. salmonicida* subsp. *salmonicida* from various geographical locations using DNA restriction fragment plasmid profiling and found the same plasmid profile to be the most common one among Danish *A. salmonicida* subsp. *salmonicida* isolates, representing 32% of the 57 investigated Danish strains in that study.

Present findings support previous results by Nielsen et al. (1993), Boyd et al. (2003), and Attéré et al. (2015) regarding high stability of plasmid pAsa1 and pAsa2 and instability of plasmid pAsa3 and pAsal1. Attéré et al. (2015) suggested an explanation for the stability of pAsa1 and instability of pAsal1 could be that pAsa1 and pAsa3 encode genes for a type II toxin-antitoxin (TA) system that kills all daughter cells that do not receive the plasmids (Boyd et al., 2003), while the TA system has not been found in plasmids pAsa2 and pAsal1 (Shao et al., 2011). Nevertheless, there is still the issue regarding stability of pAsa2 that does not encode a TA system and the instability of pAsa3 that does have a TA system (Attéré et al., 2015); an issue accentuated by the present findings. It might be possible that some clonal lineages do not acquire pAsa3 and pAsal1 (Attéré et al., 2015), which cannot be ruled out according to our results, where out of 24 *A. salmonicida* subsp. *salmonicida* that did not harbor pAsa3 and pAsal1 (plasmid profiles 15–19), 17 clustered together in four minor clades (Figure 1, four minor clades with a red ring).

Finally, it is important to stress that the present results regarding plasmids should only be taken as indications, due to the limitation of the blastn (Altschul et al., 1990) analysis that was used for creating an overview of the plasmid content. To confirm the results, additional experiments would need to be performed, such as PCR with designed primer pairs, each specific to one single plasmid.

### Genomic island profiles

Thus far, the majority of *A. salmonicida* subsp. *salmonicida* isolates from Europe have been shown to harbor either AsaGEI1b or no GEI (Emond-Rheault et al., 2015a). In this study we not only found an indication of the presence of AsaGEI1b, but also presence of a putative GEI (Table S6). Thus far in other studies, the only isolate from Europe harboring a different GEI was isolate JF3791 from Switzerland, which harbored the variant AsaGEI2b (Emond-Rheault et al., 2015b). Both AsaGEI1a and AsaGEI2a were restricted to North American isolates (Emond-Rheault et al., 2015a), while AsaGEI2c has solely been found in China (Long et al., 2016). Although it is vital to analyze more *A. salmonicida* subsp. *salmonicida* before making any conclusions, the overall general notion, is that the GEIs seem to be associated with specific geographical regions over the world and could as such possibly be used as indicators for the geographical origin of an *A. salmonicida* subsp. *salmonicida* isolate; a tool that could prove to be valuable in tracking isolates that initiate furunculosis outbreaks around the world (Emond-Rheault et al., 2015a,b; Long et al., 2016). Unfortunately, it seems that the usefulness of GEIs as indicators of the geographical origin of an isolate is limited to continents rather than countries and local regions, as there was no local geographical correlation between those isolates that seemingly harbored AsaGEI1b, the putative GEI and those who did not harbor any GEI in our results (Table S6). Though again it is important to note that the present results regarding GEIs, as with plasmids, should only be taken as indications, due to the limitation of the blastn (Altschul et al., 1990) analysis.

Interestingly 29 out of the total 33 *A. salmonicida* subsp. *salmonicida* isolates in our study that had an indication of the presence of AsaGEI1b, were grouped together in the second upper clade (Figure 1). Nine out of the fourteen *A. salmonicida* subsp. *salmonicida* that showed an indication of having a putative GEI (Table S6), were also grouped in the second upper clade. It would thus seem that AsaGEI1b or another putative GEI could have been acquired by the ancestor of the clade ~1973 (95% HPD interval 1964–1981) through a horizontal gene transfer and then passed on throughout this clade in the large clonal expansion that took place during 1975–1995 (Figure 1). Even though the GEIs contribution to the host phenotype is unknown (Emond-Rheault et al., 2015a), one could speculate that AsaGEI1b and the putative GEI provide some biological advantage to the isolates since only three out of 41 descendants in the clade (Figure 1) did not seem to harbor a GEI. This is supported by the general idea that GEIs play a crucial role in disseminating genes among bacteria that can aid in their survival and possibly increase virulence (Juhas et al., 2008).

## Conclusion

The present findings have provided novel insight into the epidemiology of the disease furunculosis revealing four main introductions in consistency with the historical expansion of the Danish aquaculture production that could have been transmitted either from freshwater to seawater or vice versa. There was also transmission of isolates harboring ARGs from a freshwater farm to seawater farms, supporting the theory of *A. salmonicida* subsp. *salmonicida* being spread from freshwater to seawater via carrier fish. The mixture of freshwater and seawater isolates from different farms in almost every minor clade and the lack of geographical connections among farms also indicates that the widespread trade of fingerlings and other fish could have played a role in the local dissemination of *A. salmonicida* subsp. *salmonicida* in Denmark. The genome based analysis moreover showed genetic and virulence variability among the highly homogenous *A. salmonicida* subsp. *salmonicida* population in Denmark, which consisted of isolates with varying plasmid and GEI profiles and plasmid encoded virulence proteins, especially those related to T3SS. In future studies it would be very interesting to include sequences of other *salmonicida* subspecies as well as other subspecies to get an idea of the broader phylogenetic distribution of *A. salmonicida* subsp. *salmonicida* and we would recommend doing a deeper analysis of plasmids and GEIs, as the purpose in this study was to create an overview of the plasmid and GEI content among the isolates and the present findings can thus only be used as indications. Overall, whole genome sequencing proved to be a highly useful tool for investigating Danish *A. salmonicida* subsp. *salmonicida* and presented important new information about this bacterium that could be relevant for possible future vaccine development.

## Author contributions

Critically revised the paper, conceived and designed the experiments: ID and FA. Analyzed the data and contributed reagents/materials/analysis tools: SB, PL, ID, and FA. Performed field sampling: ID. Performed the experiments: SB and ID. Wrote the paper: SB and PL.

### Conflict of interest statement

The authors declare that the research was conducted in the absence of any commercial or financial relationships that could be construed as a potential conflict of interest.

## Supplementary material

Figure S1 Phylogeny of *A. salmonicida* subsp. *salmonicida*. Maximum likelihood tree based on the alignment of 667 SNPs found among the 101 *A. salmonicida* subsp. *salmonicida* sequenced isolates and the reference A449. Isolates are labeled as stated in Figure 1.

Table S1 Full genomic data of the 101 sequenced *A. salmonicida* subsp. *salmonicida* isolates. Isolates are labeled as stated in Figure 1.

Table S2 Overview of 78 virulence associated and iron acquisition protein sequences found in the NCBI protein database. The following is shown in the table: name of protein sequence, short description, location of the protein coding gene, Genbank accession number and the number of *A. salmonicida* isolates harboring the given protein sequence. Isolates are labeled as stated in Figure 1.

Table S3 Overview of 17 *A. salmonicida* subsp. *salmonicida* plasmids and five genomic islands (GEI) found in the NCBI nucleotide database. The following is shown in the table: name of plasmid or GEI, short description, antibiotic resistance genes encoded on plasmid, length of plasmid or GEI (in base pairs), and Genbank accession number. Isolates are labeled as stated in Figure 1.

Table S4 Single Nucleotide Polymorphism (SNP) matrix of the 101 sequenced *A. salmonicida* subsp. *salmonicida* isolates and the reference A449. The matrix shows the SNP difference between each *A. salmonicida* subsp. *salmonicida* isolate, with 2 being the lowest and 215 being highest SNP difference among the isolates.

Table S5 Plasmid content of the 101 *A. salmonicida* subsp. *salmonicida* sequenced isolates and the reference A449. Numbers under each plasmid name represent percent coverage of that plasmid (in base pairs) for a given isolate. Except for plasmid pAsa6 and pAsa7, if percent coverage is higher than 60%, the plasmid is said to be indicated as present in the isolate and the color of the cell is green. Threshold limit for presence of plasmid in an isolate was set to 75% similarity expressed as percent sequence identity (ID) and 60% of alignment length (coverage) of the plasmid, except for plasmid pAsa6 and pAsa7 due to the high level of identity shared between pAsa5 and pAsa6 and between pAsa2 and pAsa7 respectively. Coverage was set to 100% for plasmids pAsa6 and pAsa7 in order for these plasmids being considered as present in an isolate. Following abbreviations are used for percent coverage: 100, 100% coverage; <100, from 80% up to 99% coverage; <80, from 60% up to 79% coverage; <60, from 10 up to 59% coverage; <10, from 1% up to 9% coverage; 0, 0% coverage. Isolates are labeled as stated in Figure 1.

Table S6 Genomic island (GEI) content of the 101 *A. salmonicida* subsp. *salmonicida* sequenced isolates and the reference A449. The GEI content is represented as stated in Table S5 for plasmids pAsa6 and pAsa7. Isolates are labeled as stated in Figure 1.
